# A Six-Step Model for Developing Competency Frameworks in the Healthcare Professions

**DOI:** 10.3389/fmed.2021.789828

**Published:** 2021-12-14

**Authors:** Alan Batt, Brett Williams, Jessica Rich, Walter Tavares

**Affiliations:** ^1^Department of Paramedicine, Monash University, Frankston, VIC, Australia; ^2^McNally Project for Paramedicine Research, Toronto, ON, Canada; ^3^Assessment and Evaluation, Faculty of Education, Queens University, Kingston, ON, Canada; ^4^The Wilson Centre, University of Toronto, Toronto, ON, Canada; ^5^Post Graduate Medical Education and Continuing Professional Development, Faculty of Medicine, University of Toronto, Toronto, ON, Canada

**Keywords:** competency framework, competency profile, developing competencies, professional competency frameworks, professional practice

## Abstract

Competency frameworks are developed for a variety of purposes, including describing professional practice and informing education and assessment frameworks. Despite the volume of competency frameworks developed in the healthcare professions, guidance remains unclear and is inconsistently adhered to (perhaps in part due to a lack of organizing frameworks), there is variability in methodological choices, inconsistently reported outputs, and a lack of evaluation of frameworks. As such, we proposed the need for improved guidance. In this paper, we outline a six-step model for developing competency frameworks that is designed to address some of these shortcomings. The six-steps comprise [1] identifying purpose, intended uses, scope, and stakeholders; [2] theoretically informed ways of identifying the contexts of complex, “real-world” professional practice, which includes [3] aligned methods and means by which practice can be explored; [4] the identification and specification of competencies required for professional practice, [5] how to report the process and outputs of identifying such competencies, and [6] built-in strategies to continuously evaluate, update and maintain competency framework development processes and outputs. The model synthesizes and organizes existing guidance and literature, and furthers this existing guidance by highlighting the need for a theoretically-informed approach to describing and exploring practice that is appropriate, as well as offering guidance for developers on reporting the development process and outputs, and planning for the ongoing maintenance of frameworks.

## Introduction

Competency frameworks are developed for a variety of purposes, including describing professional practice and informing education and assessment frameworks. Despite the volume of frameworks developed in the healthcare professions, and the increasing move toward competency-based education, no clear guidance exists for those who develop them (1, 2). As such, developers may be unclear about the purpose and scope of the framework, the selection of methods, and the use of such methods. This may be in part due to the lack of organizing or conceptual frameworks to guide decision making. As a result, there may be uncertainty in the appropriateness of the outputs from the development process—for example, evidence of a previous lack of focus on non-technical, structural, and teamwork competencies (3–7). These shortcomings were only reported in the years after the development and implementation of competency frameworks. To reduce some of this uncertainty, and to provide developers with a conceptual framework that is transferable across settings, we previously outlined a systems thinking approach by which to view and describe professional healthcare practice when developing competency frameworks (8). Systems thinking provides a lens that obligates a consideration of real-world contexts and complexities associated with professional practice, and the components and features required to competently enact such practice.

While a conceptual framework informed by systems thinking provides developers with an improved means by which to explore practice (8), it does little to aid developers with other, more practical elements of the development process. While guidance for developing competency frameworks exists, previous research exploring its use suggests further guidance is needed (1). Existing guidance is often vague [e.g., “use at least two methods that are complementary” (9)], and can at times be contradictory, [e.g., some suggest specific methods, while others propose it should be guided by purpose (10, 11)]. Therefore, we offer that those developing competency frameworks in the healthcare professions would benefit from renewed guidance that clarifies ambiguities in the literature, and extends guidance to include a theoretically-informed means by which to explore professional healthcare practice, and contemporary approaches to evaluation of outcomes (1, 8, 12–14). In this paper, by synthesizing and consolidating existing guidance, and leveraging recent research exploring ways of improving framework development, we will outline a six-step model for developing competency frameworks in the health professions.

## Methods

**Figure 1:**
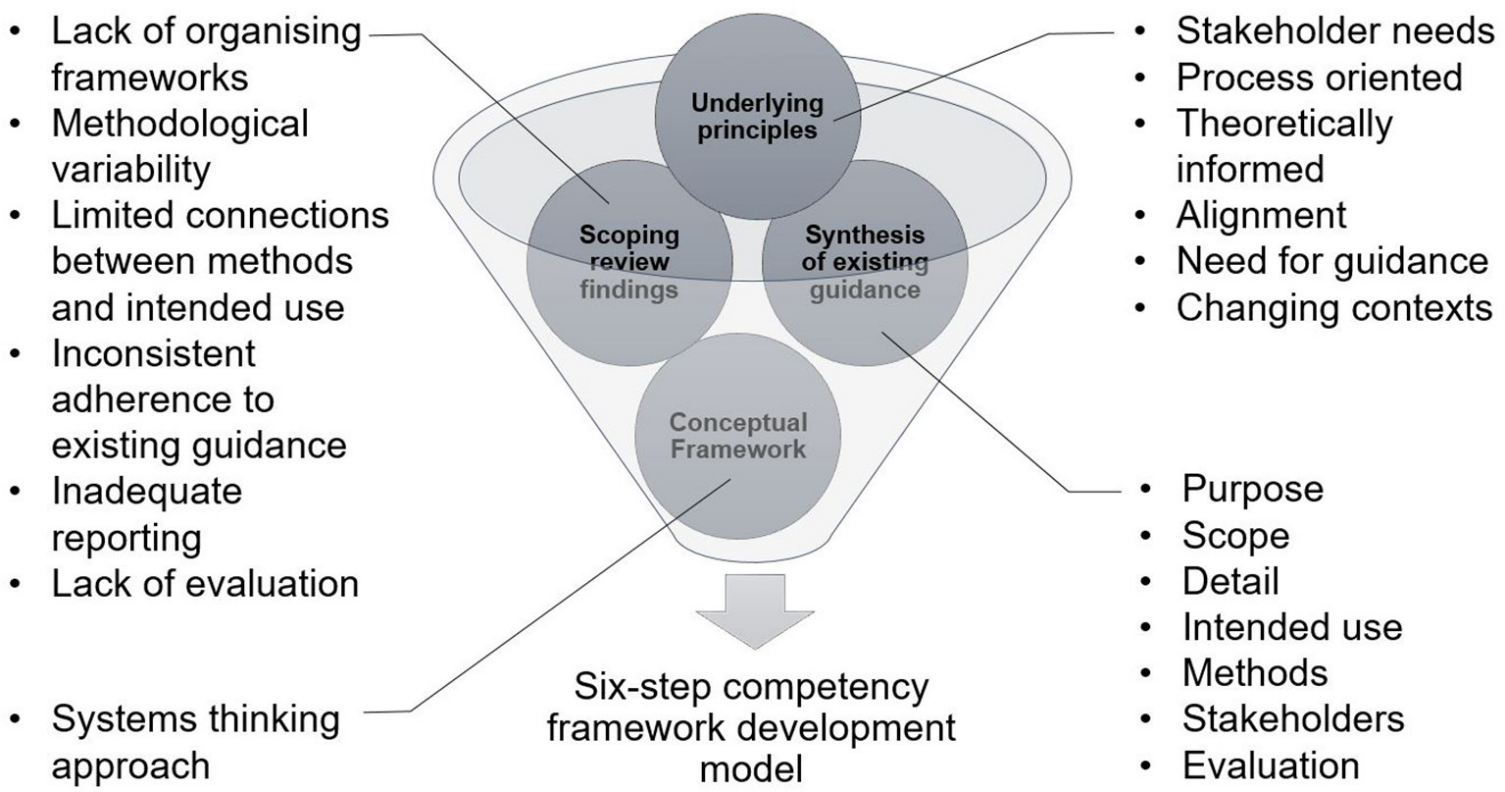
Inputs informing the structuring of guidance for the development of competency frameworks.

Using an iterative process informed by multiple sources of data we identified the necessary features of a model to provide renewed guidance for developing competency frameworks (see Figure 1 for sources). First, we utilized the findings of our scoping review of competency framework development in the healthcare professions (1). This detailed the shortcomings of the existing guidance, and highlighted several items that needed to be considered in the model. Second, we identified, organized, and synthesized the existing guidance on developing competency frameworks from multiple sources (9–11, 15–27). These sources were informed by our scoping review (1), and contemporary methodological approaches (22–27). We approached this organization and synthesis as a form of qualitative data analysis (28)—we iteratively analyzed the data, extracted the individual elements of guidance from each source, and then created categories of data from the elements (e.g., coding, and collapsing of codes). At this point, we integrated the findings from our scoping review in a similar manner. We observed a common structure in the existing guidance (e.g., plan study, gather data, translate data to competencies, report, and maintain), and elected to use this structure to inform the creation of the model. Third, we integrated a theoretically-informed approach to describing and exploring professional practice that utilized systems thinking (8). The use of a theoretical or conceptual approach to practice represents a novel contribution to the existing guidance. We incorporated this early in the model to help inform and guide subsequent choices made by developers. Finally, informed by these sources, we iteratively identified and extracted a set of underlying principles that seem to inform ways forward for renewed guidance (see Table 1).

**Table 1:** Underlying principles guiding competency framework development.

**Principle**	**Description**
Stakeholder engagement	Broad stakeholder engagement within and adjacent to the profession (e.g., end-users, regulators, educators, service providers, other healthcare professionals) informed by intended uses, purposes and targeted system(s), promotes representativeness, alignment and comprehensiveness of the developmental process and outputs (10, 11, 15).
Process oriented	Consider not just outputs, but also processes (both inputs and activities). These processes should be evaluated throughout the development, and the insights used to improve processes for ongoing competency development and revision (14, 29–31).
Theoretically informed	Theoretical approaches are required to explain how processes lead to outputs, how practice was explored and described, and how the competency framework was (or will be) evaluated (1, 8).
Alignment	While the choice of methods remains at the discretion of developers, such choices need to be aligned with a) the intended uses, purpose, and scope of the framework, and b) acceptable to the community of users (i.e., the profession) (1, 8).
Need for guidance	The development process needs broadly applicable guidelines, rather than prescriptive steps to follow, to allow for transfer across contexts and necessary adaptations for use (1).
Changing contexts	Competency frameworks are developed within dynamic health and social contexts that are subject to continuous change—specifying the competencies needed for professional practice includes accounting for this continuous change (9, 19).

## Findings

The findings of our scoping review highlighted a number of shortcomings with the existing approach to competency framework development (1). These included a focus on outputs over process, a lack of conceptual and/or theoretical frameworks, a lack of alignment between methodological choices and intended use of the framework, inconsistent adherence to existing guidance, significant variation in reporting, and a lack of planned evaluation and update of frameworks. The existing guidance on competency framework development outlined the need to identify the purpose and intended uses of the framework; the scope of contexts in which it was to be enacted; the methods used in the development process; and the stakeholders involved in the development (9–11, 15–23). A systems thinking approach to identifying and exploring practice described the need to approach practice as a patient-centered activity which occurs in dynamic health and social contexts that need to be considered. In particular, practice is increasingly inter-professional, and competencies need to reflect this (4, 32). Based on our understanding of the findings from these sources (scoping review, conceptual framework, and existing development literature), we identified a set of underlying principles to inform the development of our six-step model (see Table 1). First, there was a need for improved guidance that was not prescriptive. There needed to be alignment between methods and purpose of framework, and a renewed focus on the development process and not just the output. In line with existing guidance, broad stakeholder involvement would aid in improving the acceptability of the output to the profession (11). Finally, the model needed to acknowledge the dynamic and complex nature of practice.

**Figure 2:**
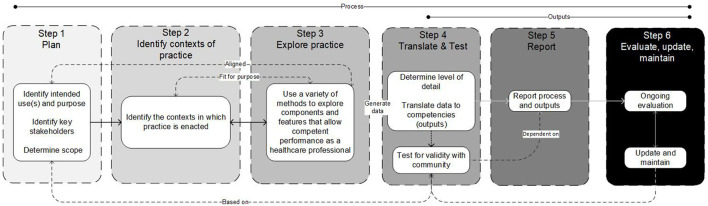
A schematic of the six-step model, illustrating the influences and connections between various steps, and indicating that the model is not necessarily intended to be implemented linearly in all cases.

The inputs informing the structuring of our improved guidance (Figure 1), suggest that any such guidance would need to account for [1] identifying intended uses, purpose, scope, detail, and stakeholders; [2] involve theoretically informed ways of identifying the people, elements, and contexts of complex, “real-world” professional practice, which includes [3] aligned methods and means by which such features and contexts can be explored. This would then provide a foundation on which to consider [4] the identification of and specification of competencies required for professional practice, [5] how to report the process and outputs of identifying such competencies, and [6] built-in strategies to continuously evaluate, update and maintain competency framework development processes and outputs (see Figure 2). Next, we will outline each step of the model. In particular, it is the inclusion of real-world contexts and complexities (8) in Step 2, and using “fit-for-purpose” aligned methods (Step 3), as well as the overall organizing framework—that we propose in this perspective— as necessary and unique augments when developing competency frameworks. We provide a practical overview of the six-step model for developers in Supplementary File 1. We provide additional evidence to highlight the utility of this model including insights derived from examples of its use in practice in Supplementary File 2.

### The Six-Step Model

#### Step 1. Plan

In Step 1 we suggest that developers consider the purpose, intended uses and scope of the framework, and identify key stakeholders and their roles. Clearly outlining the purpose (e.g., identify competencies which enable inter-professional care), intended use (e.g., to make claims about the readiness of individuals to enact those competencies), and scope (e.g., health professions A, B and C of inter-professional teams working in all public hospital settings) serves to then inform and articulate specific claims of the framework, but also to support later steps of determining whether the final output sufficiently addresses those claims (9, 33, 34). Focusing on purposes and intended uses includes whether the framework is for binary (e.g., competent/not competent, accreditation granted/not granted) or other more continuous reasons (e.g., learning). Scope refers to contexts, boundaries, underlying principles, and articulated assumptions that ultimately inform an intended time, space, and place for the framework. This helps control for unintended uses or its transferability. For example, a competency framework intended to serve complex integrated health care models carries different implications (e.g., who to include as stakeholders) than for a highly specialized role (e.g., paramedics working independently in a specific region). Stakeholders at this step—those that can productively impact the developmental process or output—serve in general, two purposes. First, to contribute to defining purpose, scope and intended uses. Second, to provide a means for developers to eventually access the system, participate in developmental decisions and evaluations of processes and outputs (described in more detail below). Stakeholders can include for example, patients, families, healthcare professionals, educators, regulators, employers and partnering health professions, and are (at least initially), defined by developers and in consideration of purpose, intended use and scope (18, 34, 35). The stakeholder composition for these activities may be different but are expected to overlap in most cases. Consideration of that composition and their involvement at this early stage provide the foundation for subsequent steps.

These early decisions, including whether it is appropriate and feasible to have multiple purposes, collectively start to inform the degree of evidence needed, the kinds of methods that will or must follow, what developmental processes may be needed, what to do when unintended uses show up (when implemented) and who has or should have a say. Other considerations such as mandate of the developer, timeframes, availability of resources (e.g., financial and manpower), development experience and expertise, maturity and state of the targeted profession, access to and the complexity of practice, and consistent terminology are expected to be discussed and influence this early stage (and subsequent stages) of the development process (1).

#### Step 2. Identify Contexts of Practice

In Step 2, developers and stakeholders are actively involved in identifying and defining the contexts in which professional practice occurs. Here we define practice as something that exists and can be defined, which involves independent and then overlapping analyses with Step 3 (9, 15, 16, 21, 36). Developers might ask: “*What is healthcare profession X?”* and “*What role does it serve the healthcare system and how is it unique?”* and “*Who does profession X interact with to serve its function?”* The intention here is to be as comprehensive as needed (given Step 1) toward understanding the professional role in context. The aim in this step is to sufficiently collect, discuss and generally be informed and influenced by the context(s) of practice. At least three different approaches can be considered. For example, developers may take iterative steps involving the analysis of existing position/profession descriptions, policy documents, or related government statements, conducting literature searches describing or informing the profession, evidence of role expansion or change, analysis of existing or planned activities, and analysis of gaps not sufficiently represented by existing competency frameworks (15). The second is to be guided by concepts derived from developmental evaluation (37). This makes evaluation an integral component of the design process, whereby inputs, processes and outputs are continuously evaluated and monitored in a rapidly changing environment. Finally, “systems thinking” has been presented as a meaningful way to structure the exploration of relevant contexts of practice (8), in order to capture the dynamic nature of contemporary healthcare systems (38, 39). This conceptual framework outlines different ways of examining the components of the system at various levels (e.g., micro, meso, macro) as well as the relationships between them (40). While other ways of approaching the system exist and what people can or should be able to do in or for that system to be deemed competent, systems thinking provides a comprehensive and flexible starting point (40–44).

Examples of methods by which to identify and explore system levels are outlined in Table 2.

**Table 2:** Examples of methods by which to identify and explore system levels.

**System level**	**Examples of methods**
Patient centred	Engage patient representatives; patient member of steering group; patient input during design and/or evaluation; other forms of patient/public engagement or involvement
Microsystem	Engage and empower those who perform the job through appropriate means (e.g., interviews, focus groups, surveys); perform job or practice analysis (16, 19)
Mesosystem	Engage other healthcare professionals (e.g., interviews, focus groups); engage and empower professional associations as stakeholders
Exosystem	Policy analysis; environmental scans, stakeholder engagement
Macrosystem	Review national/regional health policies and accords; strategic analysis of national events, government policies, agency reports
Supra-macrosystem	Review regional/international accords; identify and explore regional initiatives to collate health data, identify global forces with influence
Chronosystem	Literature reviews, review historical policy documents

#### Step 3. Explore Practice

In Step 3, developers and stakeholders are involved in exploring practice to identify the components and features that allow competent performance as a healthcare professional. Here we define practice as something people do, which involves independent and then overlapping analyses with Step 2 and eventually description (9, 15, 16, 21, 36). Developers might ask “*What does an individual in the profession do?”* and “*Does profession X perform differently depending on context?”* and “*What is it that society and end-users expect a member of profession X to be able to do?”* The intention here is to comprehensively understand how an individual in the profession enacts practice, within the context(s) identified in Step 2. Doing so will require developers to select appropriate methods to explore practice. The choice of methods by which to explore practice offers flexibility for developers (e.g., weighing practicality and cost-effectiveness, considering timeframes) (9, 15, 19, 34) but also the opportunity to support or threaten alignment/coherence goals (45) (see Table 2 for examples of methods that developers may choose to enact to identify and explore practice).

Mixing of methods or the use of multiple methods may be necessary (22, 23); however, the evidence to support the degree to which selected methods are “fit-for-purpose” should be considered with a rationale (46). Inherent in the consideration of multiple and/or mixed sources of information is the need to consider the alignment of various methods/methodologies to obtain information/data related to those sources. Selected methods should be applied defensibly, including but not limited to choice of sequence (46), and how data from multiple sources are integrated (46, 47). Developers will need to determine the representativeness of samples (e.g., do you have data to represent the perspectives of diverse stakeholders and intended users?)—this may require equity-based considerations to ensure such perspectives are represented. The blending of diverse sources of information/data is expected to require a level of interpretation by developers, which means developers will need to consider what “stake” to give each source of data. This raises considerations such as whether data sources are considered equal, the sequence of data use, the priority of sources, the merging of data, the timing of integration, and the process of analysis (47).

#### Step 4. Translate and Test

In Step 4, developers and stakeholders will work collaboratively to identify competencies informed by the data collected in Steps 2 and 3. The actual process of translating data to outputs (i.e., competencies) will be informed and guided by methodological choices. As a general approach, we suggest there is value for developers in borrowing methods from qualitative data analysis. Developers begin by exploring the data, then coding the data iteratively and inductively (28) looking for repetitive and discrepant units of meaning, collapsing codes to reduce redundancy and overlap, and generating categories of data and descriptions and key themes (i.e., competencies) from the codes. There is a level of interpretation inherent in this process, and previous guidance suggests this step is “as much art as science” (34). Developers can improve the rigor of this translation process (from data to competencies) by focusing on improving credibility (e.g., by member checking) and dependability (e.g., by clearly outlining coding methods and ensuring a detailed audit trail throughout the translation process) (28, 48).

Defensibility in this translation activity is promoted in at least three ways. These include a philosophical alignment between methods, underlying principles, contexts of practice and practice analysis; developing and implementing a plan of data collection and analysis that is methodologically defensible and fit-for-purpose; and an audit trail that can be examined by the intended community. An initial draft of the competency framework should be generated, informed by the data collected in Steps 2 and 3 (20), and developers can then engage with the broader profession, gathering feedback to further select and/or refine the framework. Healthcare professionals, subject matter experts, regulators, end-users, and those who the framework will affect as part of this translation process (10, 20, 35) should have the opportunity to reflect on the document, and provide feedback on whether it meets their needs, and reflects the values of the profession. Such a process is iterative and may require the use of consensus methods to finalize the output. This process is necessary to ensure the validity of the output (i.e., does the framework accurately represent the profession?) (35), and it should be noted that frameworks with “high-stakes” intended uses (e.g., regulation of practice) may require more extensive validation efforts in order to ensure that the output is accurate.

How a profession conceptualizes competence (e.g., degree of granularity from atomistic to holistic/integrated) will influence how developers decide to represent competence in a framework (29). Developers will need to consider the level of granularity desired in the framework (19), balancing enhanced precision (atomistic) against competency in dynamic contexts (holistic) (17, 19). Atomistic frameworks risk introducing a reductionist, decontextualized approach to complex professional practice and the assumption that the linear accumulation of items assembles neatly again to inform competence. Holistic frameworks risk being too vague or generic, ultimately threatening utility (17). The identification of an appropriate organizing or conceptual framework will guide the structure of the output. For example, developers may elect to organize competencies by roles identified and defined within the profession (e.g., roles identified in Steps 2 and 3 or existing roles).

Notwithstanding the organizing structure of the output, competencies must be considered within the context of the profession, and linked back to the intended uses, purpose, and scope (19), once the professional role is broadly understood and defined (Steps 2 and 3) (15). The output should identify and integrate the knowledge, skills, attitudes and other important attributes associated with an identifiable aspect of professional performance. Competencies should be expressed in a manner that is easily understood, recognizable, and demonstrable in professional practice (15). Informed by intended use, purpose, scope and detail, in addition to considering “what is,” developers may also wish to consider “what will be needed in the future” and “what should be” when identifying competencies (18, 19). Balancing immediate future needs (e.g., short-term developments in technology or society) with potential longer-term predictions (e.g., emerging technologies) may need to be considered.

#### Step 5. Report

In Step 5, developers report and communicate the output of the development process to the intended users and the broader profession. The six-step model outlined here, including the guiding principles, may be used to structure reporting of processes and outputs. This not only includes components of the output such as purpose, intended uses, scope etc., but also details of the processes that were undertaken—e.g., who was involved and for what purpose, how the contexts of practice were identified, how consensus was achieved, how and why methods were conducted, how data were collected and used, how the development process was evaluated throughout and how those results were used, and rationale for decisions (Step 3).

Previous efforts at reporting competency framework development largely focused on outputs, and much of the detail related to the development process remained implicit or was inconsistently reported (1). This focus on reporting of outputs means that we struggle to gain a meaningful understanding of processes and contexts, which hinders the ability to examine the validity and inherent limitations of frameworks. This can present obstacles when the community attempts to use or adopt frameworks and evaluate the short, medium, and long term outcomes of use. Finally, developers should report on the process used to translate or make sense of the data collected in Step 3 into competencies, and the results of any validation exercises (Step 4). Developers should use appropriate reporting guidelines if available and applicable.

#### Step 6. Evaluate, Update, and Maintain

Finally, in Step 6, developers plan for ongoing evaluation and updating of the competency framework (9, 15, 20) in order to ensure competencies reflect contemporary practice changes over time and to ensure their applicability and utility (19, 21). Competency framework development and outputs may be treated as a type of “program,” and evaluated using existing program evaluation techniques (4). Identifying outcome measures is key to evaluation, but developers also need to consider unintended outcomes as a result of implementation. Such unintended outcomes will only become evident through a rigorous evaluation process that considers the factors surrounding (un)successful implementation of a program and not simply whether it worked or not. For example, using a logic or program approach would require developers to look at inputs, processes, outputs, and anticipated outcomes over the short, medium and long-term, and not just whether an output was produced (30, 49). Contemporary program evaluation models emphasize the complex interactions between program factors (i.e., how and why did it work?) (12, 14, 50, 51). The use of rapid-cycle program evaluation approaches, although resource intense, may also provide developers with real-time evidence to support changes to processes and competency frameworks (13). An approach that acknowledges context, complexity and processes should be applied to evaluate the development process, outputs, and achievement of intended outcomes over time (12, 13, 30, 31, 49, 51, 52). Such approaches may help us to understand the relationships between development processes, outputs (i.e., the competencies themselves), and implementation/use of the competence framework over time, which can inform ongoing revisions/improvements to future competency development processes and frameworks.

Competency framework development is a continuous process (9, 15); thus, the reported framework or its validity is never “final.” Ongoing evaluation will help to identify what impact the framework is having, and in what areas it requires additional focus. As such, developers may wish to consider a “living document” approach whereby the framework becomes a dynamic publication that can be revisited and revised as time progresses, contexts change or practice expectations evolve. In any event, developers should form and articulate a plan to update, and maintain the competency framework over time (9, 15, 18, 19, 21, 34, 36). Previous guidance suggests that frameworks be updated every 5 years as a minimum, while acknowledging that frameworks may require more regular updates and maintenance if the profession in question is undergoing significant changes (19, 53). Thus, we refrain from proposing any criteria concerning timeframes, and instead, recommend that developers consider factors that may reflect significant changes in practice or the contexts in which it is to be enacted (e.g., technology eliminating some jobs and introducing the need for new competencies).

## Discussion

We have outlined a six-step model for developing competency frameworks in the healthcare professions that synthesizes and organizes existing guidance and literature. The six-step model advances this existing guidance by incorporating the need for a theoretically-informed approach to identifying and exploring practice. The model offers clearer guidance for developers on reporting the development process and outputs, and planning for the ongoing maintenance of frameworks. The model, despite being described as a “six-step” model, is not intended to be implemented in a stepwise linear fashion, and in practice, the competency framework development process is non-linear—earlier steps may need to be revisited as later steps are considered (2). However, while the model allows for flexibility and there is an expectation for developers to move back and forth between steps, the sequence provided is logical, and therefore using the term steps appears appropriate.

One of the claims to which this model may be vulnerable is that there is no single “correct” approach to describing or representing professional practice. Developers may wish to consider other approaches to exploring practice that may provide alternative insights. The six-step model can be adapted to allow for this. Additionally, developers using this six-step model may require significant investment in resources and time. However, the model outlined here balances obligations with intended purpose, timeline, resource availability, expected outputs and the degree of defensibility/validity called for by the community. This suggests that if the framework will be used to make high-stakes decisions (e.g., entry to practice certification), then investing in appropriate resources may be necessary. A development process informed by developmental evaluation (37), organizing frameworks, and engages those who will use the framework, may assist with validation efforts and thresholds of acceptability among the community of users. Continuous evaluation and ongoing maintenance of the framework will contribute to its ongoing utility.

We suggest that the six-step model itself is transferable across contexts and professions. We also acknowledge there may be cases whereby an output (competency framework) developed using this six-step model could be transferable to other contexts (for example developed in one country, adopted in another). However, there are risks in doing so, the main risk being the validity of the framework for use in contexts from which it was not derived (8). As a result, the framework may be missing important contextual elements of practice, thereby leading to unintended outcomes. Developers will need to determine the level of contextual similarity before adopting or adapting a framework in a novel context.

Further research should continue to apply and examine this six-step model to clarify and refine the process, improve the identification of features and components, identify additional means of data collection, and analyse and explore the process for making meaning of the data to inform usable competency frameworks. In addition, developers may find benefit in reporting guidelines, which outline the essential items that should be included when reporting research studies. The use of reporting guidelines has increased the transparency of study methods and improved the translation of study findings in other fields (54, 55).

## Conclusion

The development of competency frameworks in healthcare professions to date demonstrates variable approaches, inconsistent reporting, and inadequate descriptions of practice. We offer a six-step model intended to guide future efforts at developing frameworks. Such efforts might be improved by applying the six-step model that considers intended uses, processes, outputs, and anticipates downstream uses of the framework. The model embraces theoretically informed approaches, acknowledges context, and consolidates existing guidance. The adoption of our model facilitates the sharing of common underlying principles and therefore shifts the focus from “what” or “how many” methods were used to whether such methods were aligned with the intended purpose and overall objectives, and meet an acceptability threshold set by the community. In addition, our model accepts that change occurs over time, and competencies require continuing evaluation, maintenance, and update. This six-step model ultimately offers developers a structure by which to identify the competencies required to enact competent healthcare professional practice.

## Data Availability Statement

The original contributions presented in the study are included in the article/Supplementary Material, further inquiries can be directed to the corresponding author/s.

## Author Contributions

AB, BW, and WT devised the study. AB performed the literature review and drafted the initial manuscript. AB, BW, JR, and WT critically analyzed the literature. BW, JR and WT revised the manuscript for intellectual content. All authors contributed to the article and approved the submitted version.

## Funding

Open-access publication was funded by the Department of Paramedicine, Monash University.

## Conflict of Interest

The authors declare that the research was conducted in the absence of any commercial or financial relationships that could be construed as a potential conflict of interest. The reviewer SA declared a shared affiliation with several of the authors, AB and BW to the handling editor at the time of review.

## Publisher's Note

All claims expressed in this article are solely those of the authors and do not necessarily represent those of their affiliated organizations, or those of the publisher, the editors and the reviewers. Any product that may be evaluated in this article, or claim that may be made by its manufacturer, is not guaranteed or endorsed by the publisher.
