# Supplementary material for: A Six-Step Model for Developing Competency Frameworks in the Healthcare Professions
Source: Front Med (Lausanne). 2021 Dec 14;8:789828. doi: 10.3389/fmed.2021.789828 (PMC8713730; doi:10.3389/fmed.2021.789828)

| **Step** | **Actions** |
| --- | --- |
| **Step 1. Plan** | - Articulate the purpose of the framework - Identify the intended uses of the framework and consider the consequences of potential unintended uses - Outline the scope of the framework – the contexts and boundaries in which it is to be enacted. - Identify key stakeholders and users of the framework - Establish a representative steering group to lead the development process - Establish practical considerations – timelines, budget, available resources, access to expertise - Define consistent terminology |
| **Step 2. Identify the contexts of practice** | - Identify the contexts in which professional practice occurs - Gain an understanding of the professional role as well as the attributes required of individuals - We suggest adopting a systems perspective to identify both the contexts of practice, and the features and relationships of situated practice (practice in context) |
| **Step 3. Explore practice** | - Identify the components and features that allow for competent performance as a healthcare professional - Multiple methods or mixed-methods approaches may be necessary - Methods should be fit-for-purpose (i.e. measure what is intended), and aligned – this will be determined with reference to the intended use, purpose and scope of the framework, as well as the acceptability threshold set by the profession. - If using multiple data sources, developers will need to consider what stake to give each source – are sources equal, in what sequence are data used, the priority of sources, the merging of data, the timing of integration, and the process of analysis. - Provide a clearly outlined rationale for the use of each method - Refer to Table 2 for examples of methods to explore system levels |
| **Step 4. Translate and Test** | - Consider how the profession conceptualizes competence (atomistic, or holistic/integrated) - Consider the level of granularity desired in the framework - Competencies should be considered within the context of the profession - Competencies should be linked back to the intended uses, purpose, and scope - Identify and integrate the knowledge, skills, attitudes and other important attributes associated with an identifiable aspect of professional performance. - Competencies should be expressed in a way that is easily understood, recognisable and demonstrable in professional practice - Produce an initial list of competencies informed by the data collected in Steps 2 and 3 - Engage with the profession to select and/or refine these initial competencies based on feedback from healthcare professionals, subject matter experts, regulators, end-users, and those who the framework will affect as part of this translational process - Stakeholders should have the opportunity to reflect on the document, and provide feedback on whether it meets their needs, and reflects the values of the profession. |
| **Step 5. Report** | - Use the six-step model outlined here, including the guiding principles, to structure reporting of processes and outputs. - Report components of the output such as intended uses, purpose, scope - Report details of the processes that were undertaken – e.g. who was involved and for what purpose, how the contexts of practice were identified, how consensus was achieved, how and why methods were conducted, how data were collected and used, whether the process was evaluated throughout and how those results were used, and rationale for decisions. - Report on the process used to translate or make sense of the data into competencies, and results of any validation exercises. - Consider the use of diagrams, pictures, and heuristics to communicate competency frameworks to users - Use reporting guidelines if available and applicable. |
| **Step 6. Evaluate, update and maintain** | - Competency framework development and outputs can be treated as a type of ‘program’, and evaluated using existing program evaluation techniques - Using a logic or program approach would facilitate a look at inputs, processes, outputs, and anticipated outcomes over the short, medium and long-term, and not just whether an output was produced - The use of rapid-cycle program evaluation approaches, although resource intense, may also provide developers with real-time evidence to support changes to processes and competency frameworks - Undertake developmental evaluation throughout the development process, whereby processes and outputs are continuously evaluated and monitored in a rapidly changing environment. - Consider a ‘living document’ approach whereby the framework becomes a dynamic publication that can be revisited and revised as time progresses, contexts change or practice expectations evolve. - Form and articulate a plan to evaluate, update, and maintain the competency framework over time. - Consider factors that may reflect significant changes in practice or the contexts in which it is to be enacted to guide the timeframes for updates to the framework. - The process of updating the framework can again be guided by the six-step model. |


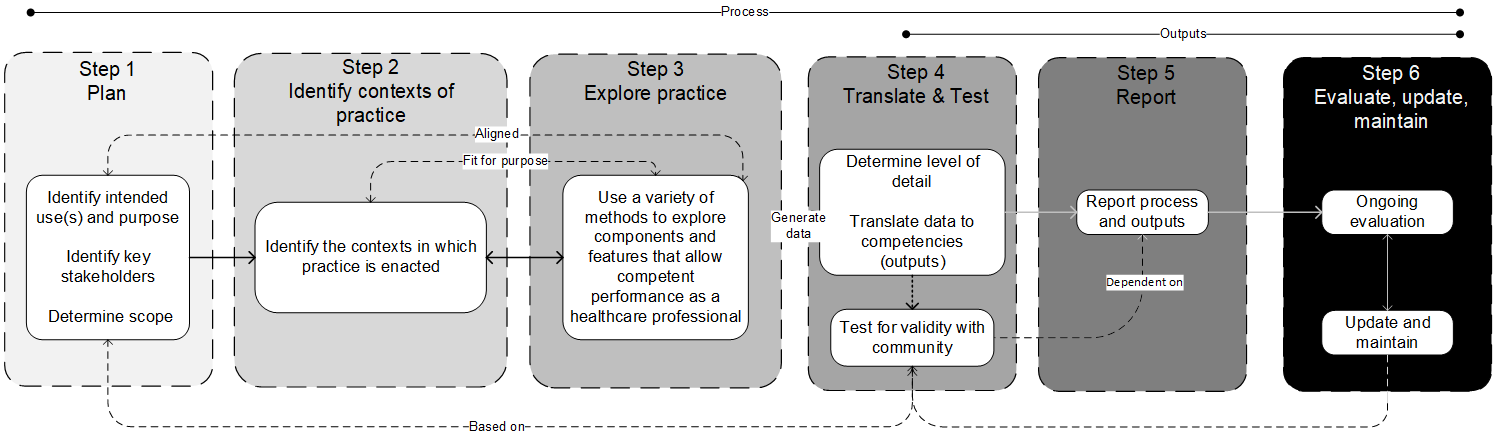

Supplement: Supplementary file 1 [file Table_1.DOCX]
