# Supplementary material for: A Six-Step Model for Developing Competency Frameworks in the Healthcare Professions
Source: Front Med (Lausanne). 2021 Dec 14;8:789828. doi: 10.3389/fmed.2021.789828 (PMC8713730; doi:10.3389/fmed.2021.789828)
Supplement: Supplementary file 2 [file Table_2.DOCX]

| **Step** | **Examples of elements** |
| --- | --- |
| **Step 1. Plan** | - Purpose: We aimed to develop a comprehensive list of quality improvement competencies for use in planning and evaluating CME programs for European general practitioners and family doctors. (1) - Intended uses: Results may help to develop undergraduate nursing curricula to facilitate nurses’ clinical application of infection control principles (2) - Key stakeholders: The Delphi panel comprised [pharmacy technicians] working in public, hospital and outpatient pharmacies, pharmacist working in similar settings with a [pharmacy technician] in their team and representatives of other stakeholders like patient organizations, insurance companies, health policy makers, the association of pharmacy professionals and all levels of pharmacy education. (3) |
| **Step 2. Identify the contexts of practice** | - While the concept of a ‘research ethics system’ has been described for over a decade, and the term continues to be used, there is no elaborated HRE system framework that sufficiently identifies the varied ways individuals can improve HRE system capacity in general or in particular contexts. Therefore, using empirical methods to delineate what comprises the HRE system would likely be necessary. (4) |
| **Step 3. Explore practice** | - Existing statements and documents provide a foundation for developing HRE competencies. These include: national and international standards for conducting human subjects research; peer-reviewed empirical and scholarly work; commentaries; sponsor statements; guidance from relevant accrediting agencies and certifying organisations (eg Accreditation of Human Research Protection Programmes, Public Responsibility in Medicine and Research, Association of Clinical Research Professionals and RCR requirements. (4) |
| **Step 4. Translate and Test** | - Example domains, subdomains and representative competencies for health research ethics education programmes are outlined by Tackett et al. (4) |
| **Step 5. Report** | - The reporting of competency frameworks in healthcare professions is outlined in detail by Batt et al. (5) |
| **Step 6. Evaluate, update and maintain** | - The competency framework evaluation was limited in that it only included pharmacist in public hospitals. If the framework is to be applicable in private and public settings and assist in defining competency required for some community pharmacies then it will require further adaptation and assessment. A more extensive evaluation and adaptation will also be needed to form a national framework due to variability of inter-state practice. (6) |

**References for examples outlined above**

1. Czabanowska K, Klemenc-Ketis Z, Potter A, Rochfort A, Tomasik T, Csiszar J, et al. Development of a competency framework for quality improvement in family medicine: A qualitative study. J Contin Educ Health Prof. 2012;32(3):174–80.

2. Liu L-M, Curtis J, Crookes PA. Identifying essential infection control competencies for newly graduated nurses: a three-phase study in Australia and Taiwan. J Hosp Infect. 2014 Feb 1;86(2):100–9.

3. Koehler TC, Bok H, Westerman M, Jaarsma D. Developing a competency framework for pharmacy technicians: Perspectives from the field. Res Soc Adm Pharm [Internet]. 2018/07/10 ed. 2018; Available from: https://www.ncbi.nlm.nih.gov/pubmed/29983262 file:///C:/Users/Alan/Documents/Mendeley Desktop/Koehler et al/Research in Social and Administrative Pharmacy/Koehler et al. - 2018 - Developing a competency framework for pharmacy technicians Perspectives from

4. Tackett S, Sugarman J, Ng CJ, Kamarulzaman A, Ali J. Developing a competency framework for health research ethics education and training. J Med Ethics. 2021 Apr 2;medethics-2021-107237.

5. Batt AM, Tavares W, Williams B. The development of competency frameworks in healthcare professions: a scoping review. Adv Health Sci Educ. 2020 Oct;25(4):913–87.

6. Carrington C, Weir J, Smith P. The development of a competency framework for pharmacists providing cancer services. J Oncol Pharm Pract. 2011;17(3):168–78.
